# Immune Lymphocyte Infiltrate and its Prognostic Value in Triple-Negative Breast Cancer

**DOI:** 10.3389/fonc.2022.910976

**Published:** 2022-07-18

**Authors:** Carlos Alexander Huertas-Caro, Mayra Alejandra Ramirez, Henry J. Gonzalez-Torres, María Carolina Sanabria-Salas, Silvia J. Serrano-Gómez

**Affiliations:** ^1^ Grupo de investigación en biología del cáncer, Instituto Nacional de Cancerología, Bogotá, Colombia; ^2^ Doctorado en Ciencias Biomédicas, Universidad del Valle, Cali, Colombia; ^3^ Facultad de Ciencias de la Salud, Universidad Simón Bolívar, Barranquilla, Colombia; ^4^ Grupo de apoyo y seguimiento para la investigación, Instituto Nacional de Cancerología, Bogotá, Colombia

**Keywords:** triple-negative breast cancer, tumor-infiltrating lymphocytes, prognosis, predictive, population groups

## Abstract

Triple-negative breast cancer (TNBC) occurs more frequently in young (<50 years) non-Hispanic black and Hispanic/Latina women. It is considered the most aggressive subtype of breast cancer, although, recently, immune infiltrate has been associated with long-term survival, lower risk of death and recurrence, and response to neoadjuvant chemotherapy. The aim of this review was to evaluate the clinical impact of the immune infiltrate in TNBC by discussing whether its prognostic value varies across different populations. A comprehensive systematic search in databases such as PubMed and Web of Science was conducted to include papers focused on tumor-infiltrating lymphocytes (TILs) in TNBC in different population groups and that were published before January 2021. TNBC patients with higher levels of TILs had longer overall survival and disease-free survival times compared with TNBC patients with low TIL levels. Similar results were observed for CD4+, CD8+ TIL populations. On the other hand, patients with high TIL levels showed a higher rate of pathological complete response regardless of the population group (Asian, European, and American). These results altogether suggest that TIL subpopulations might have a prognostic role in TNBC, but the underlying mechanism needs to be elucidated. Although the prognosis value of TILs was not found different between the population groups analyzed in the revised literature, further studies including underrepresented populations with different genetic ancestries are still necessary to conclude in this regard.

## Introduction

Breast cancer (BC) is a heterogeneous disease in its phenotypic and genomic features (1). Four intrinsic subtypes, luminal A, luminal B, HER2-enriched, and triple negative, have been reported, each one characterized by differences in the transcriptional profile and clinical behavior (2–4). The prevalence of these subtypes is variable between population groups (5, 6). Several studies have agreed that the triple-negative subtype is more prevalent in NHB and in H/L compared with non-Hispanic white (NHW) women (7–10).

Triple-negative breast cancer (TNBC) is characterized by the lack of expression of estrogen receptor (ER), progesterone receptor (PR), and human epidermal growth factor receptor 2 (HER2). It constitutes 10–20% of all breast cancers and occurs more frequently in young women (<50 years) (11, 12). It is the most aggressive subtype of BC considering that it presents with a larger tumor size and a higher histological grade at the time of diagnosis and has a high expression of cell proliferation genes, which correlated with their clinical characteristics and poor prognosis (13).

TNBC has been described as a transcriptionally heterogeneous subtype (14–16). Lehmann *et al.* (14) identified 6 subtypes through gene expression analysis: basal-like 1 (BL1) characterized by a high expression of genes involved in cell cycle and cellular division, basal-like 2 (BL2) that expresses genes that enrich the signaling by growth factors such as MET and EGFR and expresses myoepithelial markers, immunomodulator (IM) subtype that expresses genes involved in the signaling of immune cells and cytokine-mediated translation pathways, and the mesenchymal (M) and mesenchymal stem-like (MSL) subtypes which display similarities in terms of the high expression of genes involved in cell motility, epithelial–mesenchymal transition pathways, and growth factors (such as, NOTCH, PDGFR, FGFR, and TGFbeta dysregulation). However, the MSL subtype differs from the M subtype as it presents a lower expression of cell proliferation genes. Finally, the luminal androgen receptor (LAR) subtype presents a high expression of genes that participate mainly in hormonally regulated pathways, for example, by the androgen receptor (AR) (14, 17–19).

An important characteristic of TNBC is that it is the most immunogenic BC subtype. Its immune infiltrate has been associated with both the control of tumor cells and with the processes of tumor growth and metastasis (20–22). It has been likewise associated with the effectiveness of neoadjuvant and adjuvant therapy, thus correlating with the clinical outcome of the disease (23).

The variability in the immune infiltrate and its clinical impact in TNBC has been studied mainly in NHW women, but it is unknown how it may vary according to the population group. The aim of this review was to systematize those studies that have evaluated the clinical impact of the immune infiltrate in TNBC, discussing whether there are differences in its prognostic value based on the population groups.

## Tumor microenvironment and immune infiltrate In breast cancer

The neoplastic progression of BC at the cellular level depends on the interaction of the tumor microenvironment (TME) and the adjacent immune system, which can act to promote or suppress the tumor growth and invasion (24, 25).

**Figure 1:**
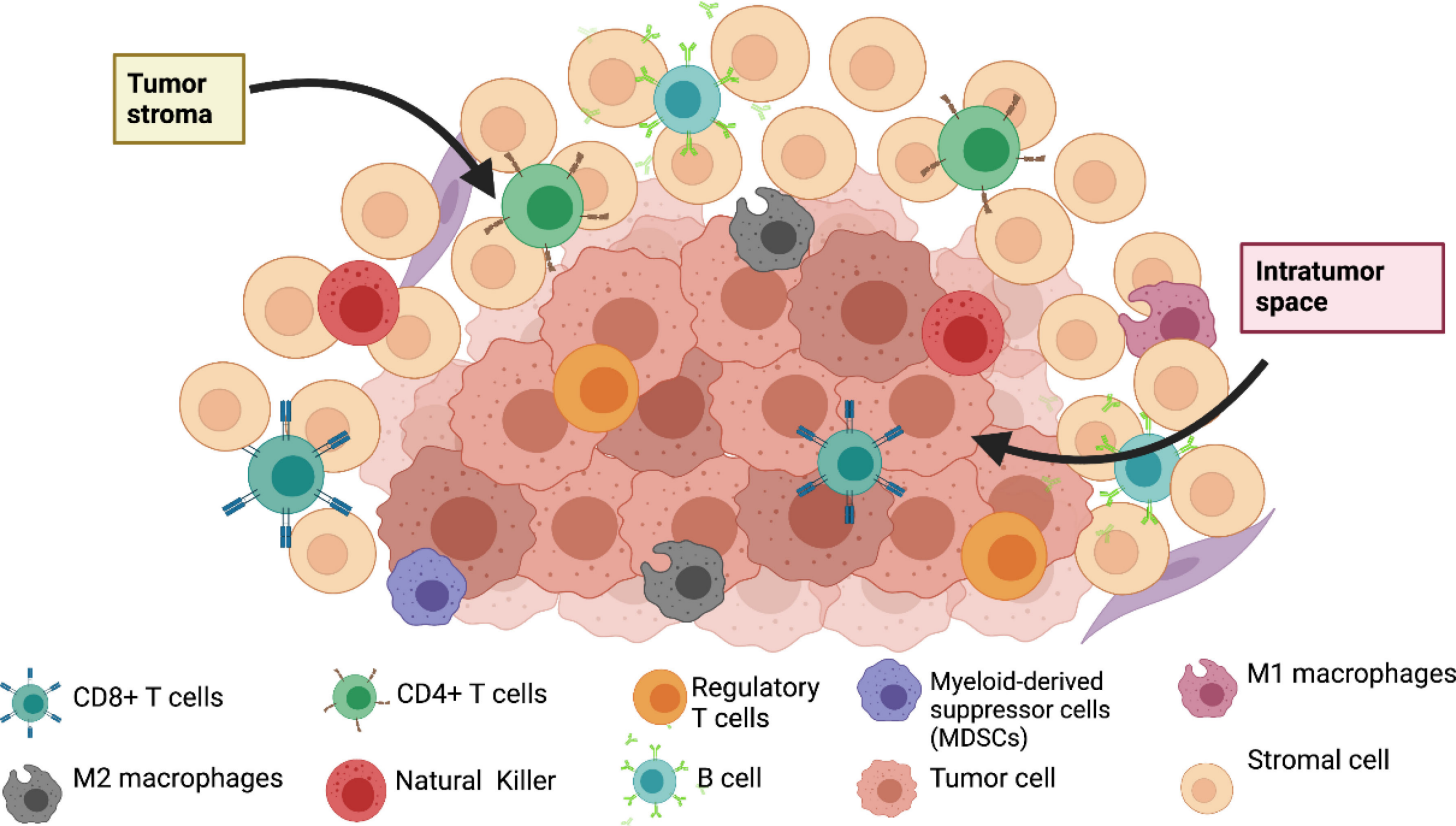
Composition of tumor microenvironment in breast cancer.

TME is composed of tumor cells and different stromal cells, such as fibroblasts, mesenchymal cells, immune cells, and adipocytes. These stromal cells secrete growth factors, cytokines, chemokines, and exosomes, molecules that maintain a constant interaction among cells within the TME (26, 27). Tumor cells are the only ones that have mutations within the TME and can promote epigenetic modifications on non-tumor cells. These modifications facilitate tumoral invasion, survival, and growth in an autocrine and paracrine way (25) (**Figure 1**).

## Composition of tumor-infiltrating lymphocytes in TNBC

The antitumor immune response in the TME is mainly driven by tumor-infiltrating lymphocytes (TILS) which, according to their location in the TME, are divided into stromal (sTILs) and intratumoral (iTILs). Most of the lymphocytes are sTILs, which infiltrate the tissue adjacent to the tumor and are considered the real tumor-infiltrating cells; on the other hand, iTILs are in direct contact with the tumor, actively infiltrating it into nests (28). It is noteworthy that different subtypes of TILs may have inhibitory or stimulatory effects on tumor progression (29)—for instance, CD8+ T cells show the highest antitumor activity that is mediated by interferon-gamma (IFN-γ), perforin, and granzyme B secretion (30). In BC, a high number of CD8+ T cells has been associated with a better prognosis and response to neoadjuvant treatment (31). On the other hand, T helper cells CD4+ have the function of enhancing the adaptive immune response by increasing the infiltration and the effector functions of CD8+ T cells and other immune cells (32). Regulatory T cells (Treg), a subpopulation of CD4+ T cells, are positive for FOXP3 and CD25 markers and participate in immune escape by suppressing the antitumor activity of CD8+ T cells (33). The presence of Treg cells within the TME is commonly associated with a poor prognosis in cancer (34). However, recent studies have demonstrated the opposite in TNBC, where the presence of Tregs in the TME was associated with longer overall survival (OS) and disease-free survival (DFS) (35, 36).

B cells can produce specific antibodies for antigens present in tumor cells; however, it has not yet been demonstrated if these cells have the same degree of clinical significance as T cells (37). The presence of B cells in the tumor stroma has been correlated with longer DFS and metastasis-free survival (MFS) in TNBC patients (38).

The role of both functionally distinct macrophage subpopulations M1 and M2 has been reported. M1 macrophages exhibit antitumoral activity by activating natural killer (NK) cells and Th1 cells (IFN- γ, IL-2, and TNF-alpha producers), which contributes to the activation of CD8+ T cells (39). In contrast, M2 macrophages or tumor-associated macrophages (TAMs) favor tumor growth and progression by facilitating tumor invasion and angiogenesis, thus being associated with a poor prognosis in patients with TNBC (40, 41).

Myeloid-derived suppressor cells (MDSCs) are a heterogeneous group of cells with immunosuppressive activity, composed mainly of granulocytes and monocytes. The MDSCs have been associated with tumoral progression through the production of immunosuppressive and pro-angiogenic cytokines that inhibit the immune response of antitumor T cells (42, 43). It should be noted that the role of MDSCs specifically in TNBC patients remains relatively unexplored (44, 45).

NK cells recognize and delete tumor cells lacking MHC-1 expression on their cell surface, whose expression is necessary for the activation of CD8 + T cells (46). Recent studies have shown that NK cells are associated with a better prognosis in the early stages of TNBC (47). More studies are needed.

## TILS as prognostic and predictive biomarker in triple-negative breast cancer

In the last few years, the predictive and prognostic role of TILs in TNBC have been studied. The relations between the composition of TILs subpopulations, clinico-pathological characteristics, and the survival of patients have likewise been explored (**Table 1**) (29, 62).

**Table 1:** Outcomes from studies that analyzed tumor-infiltrating lymphocytes (TILs) according to the region of origin.

Reference	Population	*n* (triple-negative breast cancer)	Specimen evaluated	TILs evaluated	Cut-off value	Outcomes for univariate analysis	Outcomes for multivariate analysis	Adjustment variables
(48)	Asian	308	Resected specimen	Stromal	≥10 *vs*. <10%	No specified	OS (HR: 0.493, 95% CI: 0.232–1.047)	Tumor size, LN metastasis, LVI, and histologic grade
							DFS (HR: 0.429, 95% CI: 0.215–0.859)	
(49)	Asian	61	Biopsy	Stromal	High (≥10%) *vs*. low (<10%)	For DFS (HR: 0.18, 95% CI: 0.05–0.58)	DFS (HR: 0.24, 95% CI: 0.07–0.82)	Pathological response
(50)	Asian	381	Resected specimen	Stromal	Low (<10%) *vs*. Intermediate (10–50%) + high (>50%)	RFS (HR: 2.68, 95% CI: 1.13–5.95)	RFS (HR: 2.49, 95% CI: 1.05–5.55)	Nodal status
(51)	Asian	166	Biopsy	Stromal	Continuous (per 10% increase)	pCR for sTILS (OR: 1.07, 95% CI: 1.03–1.10)	pCR for sTILS (OR: 1.05, 95% CI: 1.02–1.09)	Age, histological grade, tumor size, nodal status, LVI, Ki67 index, and NAC
				Intratumoral		pCR for iTILS (OR: 1.10, 95% CI: 1.04–1.16)	pCR for iTILs (OR: 1.06, 95% CI: 1.00–1.12)	
(52)	Asian	121	Resected specimen	Stromal	Continuous (per 10% increase)	DFS for sTILs (HR: 0.75, 95% CI: 0.28–2.03)	DFS for sTILs (HR: 0.99, 95% CI: 0.97–1.01)	Age, T stage, and nodal status
				Intratumoral		DFS for iTILs (HR: 0.66, 95% CI: 0.24–1.83)	OS for sTILs (HR: 0.99, 95% CI: 0.97–1.02)	
(53)	European	199	Biopsy	Stromal	Continuous (per 10% increase)	OS for sTILs (HR: 0.89, 95% CI: 0.78–1.02)	OS for sTILs (HR: 0.85, 95% CI: 0.74–0.99)	Grade, LN status, and treatment arm
				Intratumoral		OS for iTILs (HR: 0.83, 95% CI: 0.69–0.99)	OS for iTILs (HR: 0.82, 95% CI: 0.68–0.99)	
(54)	European	897	Resected specimen	Stromal	Continuous (per 10% increase)	DDFS (HR: 0.79, 95% CI: 0.74–0.86)	DDFS (HR: 0.76, 95% CI: 0.69–0.84)	Age, LN status, tumor size, tumor grade, peritumoral vascular invasion, and Ki67 index
						OS (HR: 0.79, 95% CI: 0.72–0.86)	OS (HR: 0.76, 95% CI: 0.68–0.84)	
(55)	European	647	Resected specimen	Stromal	≥50 *vs*. < 50%	BCFI (HR: 0.87, 95% CI: 0.79–0.95)	BCFI (HR: 0.87, 95% CI: 0.79–0.96)	Age, nodal status, tumor size, and tumor grade
						DFS (HR: 0.89, 95% CI: 0.82–0.97)	DFS (HR: 0.9, 95% CI: 0.82–0.97)	
						DRFI (HR: 0.84, 95% CI: 0.74–0.94)	DRFI (HR: 0.83, 95% CI: 0.74–0.94)	
						OS (HR: 0.83, 95% CI: 0.74–0.92)	OS (HR: 0.83, 95% CI: 0.74–0.93)	
(56)	European	607	Biopsy	Stromal	Continuous (per 10% increase)	DFS (HR: 0.93, 95% CI: 0.87–0.98)	DFS (HR: 0.95, 95% CI: 0.89–1.01)	Age, T stage, N stage, histopathological type, tumor grading, and molecular subtype
						OS (HR: 0.92, 95% CI: 0.86–0.99)	OS (HR: 0.95, 95% CI: 0.88–1.03)	
						pCR (HR: 1.16, 95% CI: 1.10–1.22)	pCR (OR: 1.17, 95% CI: 1.11–1.24)	
(57)	European	314	Biopsy	Stromal	Continuous (per 10% increase)	pCR (HR: 1.15, 95% CI: 1.05–1.26)	pCR (HR: 1.17, 95% CI: 1.06–1.30)	LPBC, tumor grade, T stage, nodal status, therapy, and age
(58)	European	304	Residual disease	Stromal	Continuous (per 10% increase)	OS (HR: 0.79, 95% CI: 0.71–0.89)	OS sTILs (HR: 0.86, 95% CI: 0.77–0.97)	Age, stage, histotype, grade, nodal status after chemotherapy, residual tumor size, neo, and neo + adj
						OS iTILs (HR: 0.78, 95% CI: 0.68–0.89)	OS iTILs (HR: 0.86, 95% CI: 0.75–0.99)	
				Intratumoral		MFS sTILs (HR: 0.79, 95% CI: 0.71–0.88)	MFS sTILs (HR: 0.86, 95% CI: 0.77–0.96)	
						MFS iTILs (HR: 0.77, 95% CI: 0.68–0.88)	MFS iTILs (HR: 0.85, 95% CI: 0.75–0.98)	
(59)	European	375	Residual disease	Stromal	Continuous (per 10% increase)	RFS (HR: 0.83, 95% CI: 0.76–0.90)	RFS (HR: 0.86, 95% CI: 0.78–0.93)	Age, pretreatment tumor size, pretreatment nodal status, and RCB class
						OS (HR: 0.82, 95% CI: 0.75–0.89)	OS (HR: 0.85, 95% CI: 0.77–0.94)	
(21)	Australian	134	Biopsy	Stromal	Continuous (per 10% increase)	DDFS (HR: 0.79, 95% CI: 0.64–0.98)	DDFS (HR: 0.77, 95% CI: 0.61–0.98)	Tumor size, histological grade, nodal status, and age
						OS (HR: 0.80, 95% CI: 0.62–1.03)	OS (HR: 0.81, 95% CI: 0.61–1.1)	
(22)	United States	481	Resected specimen	Stromal	Continuous (per 10% increase)	DRFI (HR: 0.82, 95% CI: 0.68–0.99)	DFS (HR: 0.84, 95% CI: 0.74–0.95)	Tumor size, node status, and age
						OS (HR: 0.81, 95% CI: 0.69–0.95)	DRFI (HR: 0.81, 95% CI: 0.68–0.97)	
							OS (HR: 0.79, 95% CI: 0.67–0.92)	
(60)	United States	157	Resected specimen	Stromal	Continuous	DFS (HR: 0.96, 95% CI: 0.93–1.00)	DFS (HR: 0.95, 95% CI: 0.91–1.00)	LV invasion and Nottingham histologic grade and stage
						OS (HR: 0.96, 95% CI: 0.93–1.00)	OS (HR: 0.95, 95% CI: 0.91–1.00)	
(61)	United States	605	Resected specimen	Stromal	Continuous (per 10% increase)	IDFS (HR: 0.89, 95% CI: 0.83–0.95)	IDFS (HR: 0.90, 95% CI: 0.86–0.94)	Age, menopausal status, tumor size, nodal status, Nottingham grade, Ki67 index, LPBC, histopathology subtypes, and type of breast surgery

OS, overall survival; DFS, disease-free survival; RFS, recurrence-free survival; pCR, pathological complete response; DDFS, distant disease-free survival; BCFI, BC-free interval; DRFI, distant recurrence-free interval; MFS, metastasis-free survival; IDFS, invasive disease-free survival; LN, lymph nodes; LVI, lymph–vascular invasion; NAC, neoadjuvant chemotherapy; LPBC, lymphocyte-predominant BC.

Studies carried out in Asian populations mostly showed that TILs, when evaluated in resected specimens, have a positive impact on the prognosis of TNBC (48) (50). Some studies have 95% CI with OS (HR: 0.493, 95% CI: 0.232–1.047, *p* = 0.066) when patients with high TILs (≥10%) *vs*. low TILs (<10%) were compared (48). Hida et al. (50) reported a poorer prognosis in TNBC patients with low TIL levels (<10%) compared with intermediate/high-TIL groups (>50%) (HR: 2.68, 95% CI: 1.13–5.95). This association remained significant in the multivariate model (HR: 2.49, 95% CI: 1.05–5.55). Moreover, TILs analyzed at the biopsy, before neoadjuvant chemotherapy, were found to be associated with pCR rate (*p* = 0.024). Despite previous results, opposite results have also been reported where TILs did not correlate with survival outcomes (52).

When TILs have been evaluated in biopsies, a lower likelihood of recurrence has been observed in patients with a high TIL infiltration (≥10%) compared with those with a low TIL infiltration (<10%) in univariate (HR: 0.18, 95% CI: 0.05–0.58) and multivariate analyses (HR: 0.24, 95% CI: 0.07–0.82). In addition, patients with higher TIL infiltration presented with higher pCR rates (*p* = 0.013) when compared with patients with low TIL infiltration (49) Similarly, Ruan *et al.* (51) reported a significant association between the percentage of TILs and pCR in a model adjusted for age, lymph–vascular invasion, and Ki67, both for iTILs (OR: 1.06, 95% CI: 1.00–1.12, *p* = 0.04, per 10% increase) and for sTILs (OR: 1.05, 95% CI: 1.02–1.09, *p* = 0.006, per 10% increase). When the optimal thresholds for TILs were analyzed, the results suggested that 20% is a better cutoff to determine high or low sTILs infiltration since it seems to be a better predictor of pCR (OR 2.85, 95% CI: 1.38–5.90, *p* = 0.005).

The differences in the prognosis impact of TILs between studies might be related to the clinical stage of the patients included. Presumably, there are lower amounts of tumor antigens among patients at earlier stages (31, 52), which could lead to misinterpretations regarding the relationship of TILs and clinico-pathological variables and outcomes of interest, as few studies have assessed the prognosis impact of TILs in early-stage TNBC patients.

Studies in a European population show similar findings to those in the Asian population. A study in France that evaluated TILs in the primary tumor reported a 15% reduction in the risk of death for every 10% of increase in sTIL levels (HR: 0.85, 95% CI: 0.74–0.99) and 18% reduction in the risk of death for every 10% of increase in iTILs (HR: 0.82, 95% CI: 0.68–0.99) in the multivariate analysis adjusted for the grade of lymph nodes (LN) (53).

In Italy, two studies were carried out in a larger number of TNBC patients and analyzed TILs in the resected specimen (54, 55). The first study included 897 women and reported TILs as an independent prognostic factor for a longer distant disease-free survival (HR: 0.76, 95% CI: 0.69–0.84, for every 10% increase in TILs) and longer OS (HR: 0.76, 95% CI: 0.68–0.84, for every 10% increase in TILs) in a model adjusted for age at diagnosis, lymph node stage, peritumoral vascular invasion, tumor size and grade, and Ki67 (54). The second study that evaluated sTILs in the resected specimen and dichotomized patients in having TILs ≥50% *vs*. patients with TILs <50% likewise found a 13% risk reduction in BC-free interval (HR: 0.87, 95% CI: 0.79–0.96, *p* = 0.006), 10% risk reduction for DFS (HR: 0.9, 95% CI: 0.82–0.97, *p* = 0.01), 17% for distance recurrence-free interval (HR: 0.83; 95% CI 0.74–0.94, *p* = 0.004) in a model adjusted for age, nodal status, tumor size, and tumor grade (55). A study carried out in France and Italy reported that the high presence of TILs in the residual disease after neoadjuvant treatment had a positive impact on MFS (sTIL: HR = 0.86, 95% CI: 0.77–0.96, *p* = 0.01; iTILs: HR: 0.85, 95% CI: 0.75–0.98, *p* = 0.02, per 10% increase in TILs) and longer OS (sTIL: HR: 0.86, 95% CI: 0.77–0.97, *p* = 0.01; iTILs: HR: 0.86, 95% CI: 0.75–0.99, *p* = 0.03, per 10% increase in TILs). The 5-year OS rate was 91% (95% CI, 68 to 97%) for patients with higher TILs in residual disease compared with 55% (95% CI, 48 to 61%) for patients with low TIL levels (58). Similarly, Luen *et al.* (59) found that a higher percentage of TILs in residual disease was associated with a longer recurrence-free survival (RFS) (HR: 0.86, 95% CI: 0.78–0.93, per 10% increase in TILs) and a longer OS (HR: 0.85, 95% CI: 0.77–0.94, per 10% increase in TILs).

Denkert *et al.* (56) also reported in a model adjusted for clinical parameters that patients with high TIL levels in the biopsy have longer DFS (HR: 0.93, 95% CI: 0.87–0.98, *p* = 0.011) and longer OS (HR: 0.92, 95% CI: 0.86–0.99, *p* = 0.032). However, when pCR was included in the multivariate analysis for both outcomes, the TILs were no longer significantly associated (HR: 0.95, 95% CI: 0.89–1.01, *p* = 0.11 for DFS, HR: 0.95, 95% CI: 0.88–1.03, *p* = 0.24 for OS). They also analyzed if TILs are predictors for pCR in TNBC and found a positive association for sTILs (OR: 1.17, 95% CI: 1.11–1.24, per 10% increase in sTILs). Similar results were reported by the same authors in a different study (57). A different effect of TILs according to chemotherapy regimen has been observed. TILs conferred the greatest survival benefit in patients treated with cyclophosphamide, methotrexate, and 5-fluorouracil + cyclophosphamide doxorubicin regimen (HR: 0.60, 95% CI: 0, 48 to 0.76) (54). More studies are needed to explore differences in the prognosis value of TILs according to the chemotherapy regimen.

The relationship between higher TIL levels and higher pCR rates could be explained by the degree of antitumor immune response by TILs against cancer cells that act synergistically with the natural-immunity-restoring antitumor response (20, 22). In addition, it has been demonstrated that chemotherapy treatment can promote the antitumor immune response due to the production of danger signals—danger-associated molecular patterns—during cell death. The expression of calreticulin (CALR) and release box 1 of the high mobility group (HMGB1) also boosts this antitumor immune response (63). All these could be together related to the presence of TILs in residual disease (58), and thus a good prognosis was reported for TILs in residual disease (64).

In the Australian population, an analysis that included early-stage TNBC patients showed that for every 10% increase in the presence of TILs in the primary tissue, there was a 13% decrease in the risk of distant relapse (HR: 0.77, 95% CI: 0.61 –0.98, *p* = 0.02) in a model adjusted for clinico-pathological characteristics. No statistically significant differences were observed for OS (21).

In the United States, Adams and colleagues (22) reported that for every 10% increase in sTILs evaluated in surgical specimens, there was a 16% reduction in the risk of recurrence (HR: 0.84, 95% CI: 0.74–0.95, *p* = 0.005) and a 21% reduction in the risk of death (HR: 0.79, 95% CI: 0.67–0.92). In the same direction, Krishnamurti and colleagues (60) showed that higher peripheral TILs were associated with a better survival (HR: 0.95, 95% CI: 0.91–1.00, *p* = 0.0354) and less chance of recurrence (HR: 0.95, 95% CI: 0.91–1.00, *p* = 0.0314). Leon-Ferre *et al.* (61) reported a similar association between sTILs and invasive disease-free survival in patients with TNBC diagnosed at early stages (HR: 0.90, 95% CI: 0.86–0.94, per 10% increment in TILs).

The case-only study that includes 86 Peruvian women with TNBC observed a statistically significant association between TIL density and a higher tumor grade (*p* = 0.006), but no significant association was found regarding the relationship between sTILs and survival (65). More studies are needed in the Latino population.

## The subpopulation of TILS and its prognostic value

Due to the relevance of TILs in TNBC, in recent years, an attempt has been made to elucidate the role of the different TIL subpopulations, in particular, the most recurrent ones such as CD8, CD4, and FOXP3 (**Table 2**).

**Table 2:** Outcomes from studies that analyzed tumor-infiltrating lymphocytes (TIL) subpopulations according to the region of origin.

Reference	Population	*n* (triple-negative breast cancer)	Specimen evaluated	Biomarker analyzed	Outcomes for univariate analysis	Outcomes for multivariate analysis	Adjustment variables	Methodology
(66)	Asian	39	Biopsy and residual disease	CRF	CRF low *vs*. high	CRF low *vs*. high	Pathological response	Tissue sections
					RFS (HR: 11.420, 95% CI: 2.215–208.742)	RFS (HR: 13.021, 95% CI: 2.241–258.136)		
					OS (HR: 9.847, 95% CI: 1.883–180.764)	OS (HR: 8.346, 95% CI: 1.538–155.128)		
(67)	Asian	164	Biopsy	CD8	None reported	CD8 iTILs high *vs*. low	Tumor size, LN stage	TMA
						DFS (HR: 0.48, 95% CI: 0.27–0.83)		
						OS (HR: 0.59, 95% CI: 0.32–1.07)		
				CD4		CD4 iTILs high *vs*. low		
						DFS (HR: 0.62, 95% CI: 0.36–1.07)		
						OS (HR: 0.55, 95% CI: 0.30–1.01)		
						CD4 sTILs high *vs*. low		
						DFS (HR: 0.46, 95% CI: 0.26–0.82)		
						OS (HR: 0.44, 95% CI: 0.24–0.83)		
(68)	Asian	110	Biopsy	CD8	CD8/FOXP3 (high *vs*. low)	CD8/FOXP3 (high *vs*. low)	Age, menopausal status, tumor size, TNBC subtype, Ki67, CD8, and VPR	Tissue sections
				FOXP3	pCR (HR: 4.93, 95% CI: 1.82–15.09)	pCR (HR: 5.32, 95% CI: 1.62–19.98)		
(35)	Asian	164	Biopsy	Treg	Intratumoral Treg (high *vs*. low)	Intratumoral Treg (high vs. Low)	Tumor size, nuclear grade, and age	TMA
					OS (HR: 0.59, 95% CI: 0.33–1.04)	OS (HR: 0.49, 95% CI: 0.25–0.95)		
					DFS (HR: 0.49, 95% CI: 0.20–0.83)	DFS (HR: 0.33, 95% CI: 0.17–0.66)		
(69)	Asian	278	Resected specimen	FOXP3	Stromal FOXP3 (high *vs*. low)	Stromal FOXP3 (high *vs*. low)	TNM stage, p53 status, EGFR status, Scd8, TILs, Sfoxp3, and prognostic risk score	Tissue sections
					OS (HR: 1.743, 95% CI: 1.111–2.734)	OS (HR: 1.712, 95% CI: 1.085–2.702)		
(70)	European	179	Resected specimen	CD8	High *vs*. low	High *vs*. low	Tumor size	Tissue sections
					OS (HR: 2.1, 95% CI: 1.1–4.5)	OS (HR: 1.8, 95% CI: 1.1–4.4)		
(71)	European	213	Biopsy	TILs	None reported	Average TILs	CD3, CD8, FOXP3, CD20, and CD68	Tissue sections
						BCSS (HR: 0.3, 95% CI: 0.1–0.8)		
(72)	European	175	Resected specimen	FOXP3	None reported	High *vs*. low	N/A	TMA
						RFS (HR: 0.371, 95% CI: 0.213–0.644)		
						DSS (HR: 0.416, 95% CI: 0.231–0.750)		
(73)	United States	183	None specified	FOXP3	High *vs*. low	None reported	N/A	TMA
					OS (HR = 12.7, 95% CI: 4.5–35.6)			
				CD163	High *vs*. low			
					OS (HR = 3.2, 95% CI: 1.7–6.2)			
(74)	United States	160	Resected specimen	CD8	High *vs*. low in AA	High *vs*. low in AA	Age	TMA
					OS (HR: 0.51, 95% CI: 0.25–1.03)	OS (HR: 0.51, 95% CI: 0.25–1.04)		

OS, overall survival; DFS, disease-free survival; RFS, recurrence-free survival; pCR, pathological complete response; BSCC, BC-specific survival; LN, lymph nodes; AA, African American.

A study conducted in the Asian population in which the number of TILs CD8+ and TILs FOXP3+ was analyzed in biopsy and residual tissue reported that a high rate of change in the CD8+/FOXP3+ ratio was an independent prognostic factor for recurrence and survival (66). In a different study, high levels of iTILs CD8+ were associated with DFS (HR: 0.48, 95% CI: 0.27–0.83) but not with OS (HR: 0.59, 95% CI: 0.32–1.07). On the other hand, patients with higher levels of sTILs CD4+ presented longer DFS (HR: 0.46, 95% CI: 0.26–0.82) and OS (HR: 0.44, 95% CI: 0.24–0,83) (67). Regarding clinico-pathological variables, a correlation between the immune infiltrate and age at diagnosis has also been reported. The highest rates of the CD8+/FOXP3+ ratio were observed more frequently in women diagnosed at an early age (*p* = 0.003), specifically when they are still in a premenopausal state (*p* = 0.002) (68). Moreover, a high CD8+/FOXP3+ ratio was found as a strong predictor of pCR (OR: 5.32, 95% CI: 1.62 to 19.98) (68).

Studies in less common subpopulations, such as B-cell (CD20+) and Tregs (FOXP3+/CD3+), have found them positively associated to better outcomes. A Kaplan–Meier analysis showed that patients with higher intratumoral Treg presented longer DFS (*p* = 0.001). A multivariate analysis confirmed this association (HR: 0.33, 95% CI: 0.165 to 0.659). High intratumoral Treg infiltration was also found to be associated with OS (HR: 0.49, 95% CI: 0.25–0.95). Additionally, patients with higher CD20+ B-cell infiltration in both the intratumoral (DFS: *p* = 0.015; OS: *p* = 0.020) and stromal (DFS: *p* = 0.012; OS: *p* = 0.031) compartments presented better clinical outcomes (35). Tian and colleagues (69), in a Chinese study, categorized patients according to the DFS times and reported that patients in the DFS ≥5 years group had higher NK cell stromal infiltration (*p* < 0.001) and low stromal TAM infiltration (*p* = 0.004). Stromal FOXP3+ TILs were found as an independent prognostic factor for OS (sTILs FOXP3+ low/high HR: 1.712, 95% CI: 1.085–2.702) (69).

Regarding the studies in a European population, it was observed that patients with low TIL CD8+ infiltration were associated with a higher risk of death from BC (HR: 2.2, 95% CI: 1.0–3.8) (70). On the contrary, Althobiti and colleagues (71) only found TILs as an independent predictor of good prognosis in a model that included various immune cells, such as CD3, CD8, FOXP3, CD20, and CD68. West and colleagues (72) reported that a high infiltration of TILs FOXP3+ was strongly associated with better outcomes (RFS: HR = 0.371, 95% CI: 0.213–0.644; *p* = 0.0004) and disease-specific survival (HR = 0.416, 95%: CI 0.231–0.750; *p* = 0.0036). In contrast, a study from the United States reported that a high expression of FOXP3 and CD163 was associated to a worse OS (HR = 12.7, 95% CI: 4.5–35.6 and HR = 3.2, 95% CI: 1.7–6.2, respectively) (73).

Few studies have analyzed the differences in the tumor microenvironment between European American (EA) women and African American (AA) women, and the results have been contradictory. Preliminary data from Wright and colleagues (75) found higher levels of TILs in early-stage (I–II) tumors from AA patients compared with EA (*p* = 0.019), but this difference was not observed for late-stage (III–IV) tumors. TILs also correlated negatively with AR expression and positively with PD-L1 expression. The analysis of CD8+ T cell infiltration in AA and EA women revealed that AAs with high CD8 infiltration have a trend towards better survival compared with AA with low CD8 infiltration (HR: 0.51, 95% CI: 0.25–1.04) (74). On the other hand, a study that analyzed The Cancer Genome Atlas database and compared the immune gene expression between AA and EA women did not find large-scale immunogenic differences (76).

TILs have a useful prognostic role in TNBC based on TIL populations. Nevertheless, the immune infiltrate phenotype and its prognostic value require better understanding. Thus, it is necessary to include other immune cell populations in future studies. The association reported between the high Treg FOXP3 infiltrate and better DFS and OS in TNBC is interesting considering that Treg has been associated with a poor prognosis as it can suppress antigen-presenting cells and other immune cells, events that are regulated through the secretion of inhibitory cytokines, granzyme B, and perforin (77). On the contrary, the favorable prognosis may be explained by the positive correlation between FOXP3 infiltration and TILs CD8+ infiltration (68). There is a need to clarify the prognostic role of Treg FOXP3+ in TNBC tumors.

## Expression of membrane markers in the immune infiltrate

In addition to the different immune cell’s populations mentioned before, there are other biomarkers of special interest, such as the expression of PD-L1. Studies in different populations have consistently showed a correlation between a high expression of PD-L1 in tumor cells and higher levels of sTILs (78–80).

Regarding the impact of PD-L1 in a patient’s prognosis, controversial results have been published. A study from Japan found PD-L1 positive/TILs low expression as an independent negative prognostic factor for RFS (HR = 4.7, 95% CI: 1.6–12.7) and OS (HR = 8.4, 95% CI: 2.3–30.3) (79). AiErken and colleagues (80) conducted a study that included Chinese patients diagnosed with TNBC and reported a positive PD-L1 expression as an independent prognosis factor for OS (HR: 0.302, 95% CI: 0.127–0.721) and DFS (HR: 0.451, 95% CI: 0.211–0.963). A study from the United States reported that elevated levels of PD-L1 were associated with decreased OS compared with a low expression (HR: 10.4, 95% CI: 3.6–29.6) (73). On the contrary, Li and colleagues found that any stromal PD-L1 expression was associated with better DFS but not OS (81).

The association between the expression of PD-L1 and a high percentage of TILs could be explained by activated T cells, which produce IFNγ (82). It has been proposed that IFNγ induce PD-L1 expression as an immune evasion mechanism by the tumor (83). Additionally, the relationship of high TIL levels and PD-L1 expression could also explain the association between PD-L1 expression and DFS and OS in Asian populations (83) and the pCR rates in European populations (80).

Cerbelli *et al.* (78) analyzed 54 TNBC biopsies taken from different institutions in Rome, Italy, and found a statistically significant association between PD-L1 expression in ≥25% of neoplastic cells and pCR (OR: 1.13, 95% CI: 1.01–1.27). Additionally, it was observed that 100% of the patients who achieved a pCR presented jointly a higher percentage of TILs and PD-L1 expression in ≥25% of tumor cells (*p* = 0.011). These results suggest that PD-L1 expression could be a marker of response to neoadjuvant chemotherapy in patients with TNBC. However, to reach this conclusion, more and larger studies that focus on the expression of PD-L1 in TNBC patients treated or not with neoadjuvant chemotherapy are needed—for instance, PD-L1 is described to be more commonly expressed in primary tumors than metastatic tumors (*p* = 0.002) (84), although some controversial results have also been published (85).

TIM3 is an immune checkpoint molecule that is expressed on CD4+ helper 1 (Th1) cells, CD8+ T cells, dendritic cells, and other subpopulations of lymphocytes, macrophages, and monocytes (86). The high expression of PD-1 and PD-L1 was each associated with a high expression of TIM-3 (*p* = 0.0001 and *p* = 0.0019, respectively). Patients with a higher TIM-3 expression presented better DFS (HR: 0.1072, 95% CI: 0.0319–0.3603) and longer OS (HR: 0.1129, 95% CI: 0.0323–0.3948) (86).

Interestingly, a German study analyzed the expression levels of 12 immune genes that included T cells, B cells, cytokines, and immune checkpoints markers (CXCL9, CCL5, CD8A, CD80, CXCL13, IGKC, CD21, IDO1, PD-1, PD -L1, CTLA4, and FOXP3). Based on their gene expression, they categorized the patients in three immune groups: low expression (A), intermediate expression (B), and high expression (C). They observed differences in the pCR rates among the three groups: 24% for A, 37.4% for B, and 50.4% for C (*p <*0.001). All 12 immune genes at the mRNA level were significantly linked to pCR; the best predictors were PD-L1 (OR: 1.44, 95% CI: 1.18 to 1.77, per ΔCt) and CD80 (OR: 1.74, 95% CI: 1.28 to 2.38, per ΔCt) (57).

## Conclusions

Although it is not doubted that TILs play an essential role in tumor development, the methods used across studies to measure the infiltrate are heterogeneous (87)—for example, it has been recommended to consider as high an infiltration value >50% (88) or a cutoff point >60% (89) or even to consider three cutoff points (<10%, between 10 and 50%, and >50%) (90). Moreover, studies differ in their sample sizes and inclusion criteria. Some studies evaluate TILs in biopsies and others in the resected specimens of patients that received neoadjuvant chemotherapy or not. Other studies included only early-stage patients. Therefore, all these circumstances make it difficult to provide an assertive comparison between studies to conclude on the role of TILs in carcinogenesis.

The classification of triple-negative breast cancer by immunohistochemical techniques could also be a source of heterogeneity. As mentioned above, some studies included biopsies and others resected specimens. The heterogeneity in the expression of immunohistochemistry markers such as ER, PR, and HER2, when evaluated in core needle biopsies or in a resected specimen, could lead to the misclassification of breast cancer into intrinsic subtypes (91–93). We cannot rule out that there may be misclassified cases among studies and that this may explain, in part, why some studies did not find statistically significant differences in some of the outcomes evaluated. It is also important to consider if TILs were evaluated in resected specimens from patients who previously received neoadjuvant chemotherapy since it is well known that chemotherapy can modify the panorama of the immune infiltrate, and this could impact the results of TIL characterization (94–96).

Germline *BRCA1/2* mutations range between 9 and 21% in unselected TNBC patients (97, 98). The presence of mutations in repair genes could lead to a greater formation of neoantigens, which would translate into an increase in immune infiltrate in these cases (99–102). For this reason, it is important to analyze the results of the studies considering the germinal component to avoid bias in the results.

In any case, the results presented below on the prognostic and predictive value of TILs in different populations such as Asian, European, Australian, and American present similar risk directions highlighting that TILs might be an independent prognostic factor for recurrence and survival and an independent predictor factor for pCR regardless on the origin of the patients.

## Author Contributions

Writing—review of the draft: CH-C, MR, and HG-T. Conception and study design: SS-G, CH-C, and MS-S. Manuscript preparation: CH-C, MR, HG-T, and SS-G. Writing—reviewing and editing: SS-G and MS-S. All authors contributed to the article and approved the submitted version.

## Funding

This work was supported by Minciencias (Contrato 838-2018 to SS-G) and the Colombian National Cancer Institute (C-19010300431 to SS-G).

## Conflict of Interest

The authors declare that the research was conducted in the absence of any commercial or financial relationships that could be construed as a potential conflict of interest.

## Publisher’s Note

All claims expressed in this article are solely those of the authors and do not necessarily represent those of their affiliated organizations, or those of the publisher, the editors and the reviewers. Any product that may be evaluated in this article, or claim that may be made by its manufacturer, is not guaranteed or endorsed by the publisher.
